# CCR4 Blockade Diminishes Intratumoral Macrophage Recruitment and Augments Survival of Syngeneic Pancreatic Cancer-Bearing Mice

**DOI:** 10.3390/biomedicines11061517

**Published:** 2023-05-24

**Authors:** Aydar Khabipov, Dung Nguyen Trung, Julia van der Linde, Lea Miebach, Maik Lenz, Felix Erne, Wolfram von Bernstorff, Tobias Schulze, Stephan Kersting, Sander Bekeschus, Lars Ivo Partecke

**Affiliations:** 1 Department of General, Thoracic, Visceral, and Vascular Surgery, Greifswald University Medical Center, Ferdinand-Sauerbruch-Str., 17475 Greifswald, Germany; 2 ZIK *plasmatis*, Leibniz Institute for Plasma Science and Technology (INP), Felix-Hausdorff-Str. 2, 17489 Greifswald, Germany; 3 Clinic and Policlinic for Dermatology and Venerology, Rostock University Medical Center, Strempelstr. 13, 18057 Rostock, Germany; 4 Department of General, Visceral, and Thoracic Surgery, Helios Clinic Schleswig, St. Jurgener Str. 1–3, 24837 Schleswig, Germany

**Keywords:** CCL17, CCL22, M2 macrophages, migration, TAMs, tumor-associated macrophages

## Abstract

Pancreatic cancer is known for its tumor microenvironment (TME), which is rich in stromal and immune cells supporting cancer growth and therapy resistance. In particular, tumor-associated macrophages (TAMs) are known for their angiogenesis- and metastasis-promoting properties, which lead to the failure of conventional therapies for pancreatic cancer. Hence, treatment options targeting TAMs are needed. The C-C chemokine receptor type 4 (CCR4) is critical for immune cell recruitment into the TME, and in this paper we explore the effects of its genetic or immunotherapeutic blockade in pancreatic-cancer-bearing mice. Murine PDA6606 pancreatic cancer cells and murine peritoneal macrophages were used for in vitro migration assays. In vivo, a syngeneic, orthotropic pancreatic cancer model was established. Tumor growth and survival were monitored under prophylactic and therapeutic application of a CCR4 antagonist (AF-399/420/18025) in wildtype (CCR4^wt^) and CCR4-knockout (CCR4^−/−^) mice. Immune infiltration was monitored in tumor tissue sections and via flow cytometry of lysed tumors. PDA6606 cells induced less migration in CCR4^−/−^ than in CCR4^wt^ macrophages in vitro. Pancreatic TAM infiltration was higher, and survival was reduced in CCR4^wt^ mice compared to CCR4^−/−^ mice. Antagonizing CCR4 in wildtype mice revealed similar results as in CCR4^−/−^ mice without antagonization. Prophylactic CCR4 antagonist application in wildtype mice was more efficient than therapeutic antagonization. CCR4 seems to be critically involved in TAM generation and tumor progression in pancreatic cancer. CCR4 blockade may help prolong the relapse-free period after curative surgery in pancreatic cancer and improve prognosis.

## 1. Introduction

C-C chemokine receptor type 4 (CCR4/CD194) and its ligands CCL17 and CCL22 are fundamentally involved in the migratory processes of immune cells of the innate and adaptive immune systems [1,2,3]. The pro-tumorous role of CCR4 in the progression of T-cell-associated lymphoma is well known and explained by a higher infiltration with tumor-promoting T-cell subpopulations, such as regulatory T cells (T_regs_) and T_H_2 cells, which both show an increased expression of CCR4 [4,5,6]. In addition, many cancer entities enable the recruitment of naive immune cells and induce their differentiation into a pro-tumorous phenotype. A prominent example of a distinct influence of cancer on immune cells is pancreatic ductal carcinoma (PDA) [7]. PDA is one of the deadliest forms of cancer, with a five-year survival rate of less than 10% [8]. PDA is characterized by the accumulation of genetic alterations promoting malignant pancreatic cell transformation. The most commonly mutated gene in PDA is K-Ras, which is present in up to 95% of PDA cases [9,10]. The K-Ras mutation results in the constitutive activation of downstream signaling pathways, including the mitogen-activated protein kinase (MAPK) and phosphatidylinositol-3-kinase (PI3K)/Akt pathways, which promote cell survival, proliferation, and migration [11,12].

In addition to K-Ras, PDAC is characterized by a complex interplay of genetic alterations and cellular signaling pathways contributing to disease progression. These alterations include mutations in tumor-suppressor genes such as TP53, CDKN2A, and SMAD4, which can result in the loss of normal cellular functions and promote tumor growth and metastasis [13,14]. Other genetic alterations—such as amplifications of MYC and KRAS, or deletions of PTEN and SMAD4—have also been reported in PDAC [15,16]. These genetic alterations and signaling pathways interact intimately with stromal and immune cells, forming a growth-supporting tumor microenvironment (TME) [17,18]. The TME comprises various stromal and immune cells, including cancer-associated fibroblasts, immune cells, and extracellular matrix components. In particular, the interactions between pancreatic cancer cells and immune cells—such as regulatory T cells (T_regs_) and tumor-associated macrophages (TAMs)—contribute to therapy resistance and poor outcomes in pancreatic cancer patients [19,20].

This study investigates the CCR4-associated recruitment of immune cells—especially TAMs—to the TME. TAMs share many similarities with anti-inflammatory M2 macrophages, which promote angiogenesis, tumor growth, and metastasis [21,22,23,24,25]. It has been shown that PDA can differentiate monocytes to tumor-associated macrophages (TAMs) [26,27,28]. Accordingly, as experimental evidence suggests, TAM inhibition is a promising strategy as a complement to adjuvant therapy [29,30]. In addition to CCL17 and CCL22, CCL2 (monocyte-chemo-attractant protein 1, MCP-1) critically drives TAM formation [31,32], and its receptor CCR2 is involved in cancer progression [33,34,35]. In a phase Ib clinical trial in cases of borderline resectable and locally advanced pancreatic cancer, PF-04136309—an orally available small-molecule CCR2 inhibitor—significantly reduced bone marrow monocyte recruitment into peripheral blood. Receptor blockade by an orally administered selective CCR2 inhibitor (CCX872-B) and ligand blockade by a humanized monoclonal CCL2-neutralizing antibody (CNTO 888) in clinical trials were therefore expected to be promising. Surprisingly, these trials did not provide the anticipated benefits regarding tumor growth reduction, metastasis inhibition, and long-term survival in solid tumors [36,37,38]. Furthermore, the interruption of CCL2 inhibition in a breast cancer model was followed by a rebound of circulating monocytes [39], leading to increased angiogenesis, metastases, and death in tumor-bearing mice.

By better understanding the genetic alterations and signaling pathways that contribute to PDAC’s development and triggers of a pro-tumorous microenvironment, more effective strategies to target these pathways and improve patient outcomes ca be developed. In this matter, it is necessary to identify modalities for inhibiting TAM precursor cell migration. Besides CCR2, CCR4 has been reviewed and recognized as a highly relevant receptor for immunological tumor infiltration [28]. Interestingly, CCR4 can also bind monocyte chemoattractant protein 1 (CCL2) [40,41], and macrophages have been reported to express CCR4 [31,32]. Furthermore, CCR4 is crucial for macrophage migration [33] and, therefore, is a potential immunological target for suppressing TAM formation. This study examined the impact of genetic (CCR4^−/−^) and prophylactic or therapeutic pharmacological CCR4 blockade on TAM infiltration, pancreatic cancer growth, and animal survival in a murine orthotopic pancreatic cancer model.

## 2. Materials and Methods

### 2.1. Cell Culture

The murine cell line 6606PDA (Pancreatic Ductal Adenocarcinoma) was a kind gift from Prof. David Tuveson (Cancer Center at Cold Spring Harbor Laboratory, Cold Spring Harbor, NY, USA). The cells were originally isolated from pancreatic adenocarcinoma of a transgenic C57BL/6 mouse strain bearing the KrasD12G allele. Cell culture was performed in dishes containing Roswell Park Memorial Institute (RPMI) 1640 medium supplemented with 10% fetal bovine serum and 100 μg/mL penicillin/streptomycin (all Sigma-Aldrich, Taufkirchen, Germany), under standard cell culture conditions.

### 2.2. In Vivo Experiments

Six-to-twelve-week-old male CCR4^−/−^ mice (C57BL/6 background) and male wildtype mice (C57BL/6^wt^) were used for the orthotopic, syngeneic pancreatic cancer model, as previously established by our group [42,43]. For pancreatic cancer implantation, the abdominal cavity was opened with a 1.5 cm transverse incision after anesthesia by intraperitoneal application of a mixture of Ketanest S and Rompun (Pfizer, Berlin, Germany). Induction of pancreatic cancer was realized by orthotropic injection of 2.5 × 10^5^ 6606PDA cells suspended in 10 μL of fully supplemented cell culture medium and 10 μL of Matrigel into the pancreatic head. The abdomen was closed with continuous sutures. Subcutaneously injected buprenorphine (0.1 mg/kg) was used for postoperative pain management. Our group identified postoperative complications based on a stress score in mice with pancreatic cancer [44]. For tumor growth analysis and histological examination, three different in vivo experiments were performed: First, pancreatic-cancer-bearing CCR4^−/−^ mice were compared to cancer-bearing wildtype mice. Each group initially contained 15 animals. Second, prophylactic, intraperitoneal administration of a CCR4-antagonizing small molecule (1.5 µg of AF399/420/18,025 in 250 µL of dimethyl sulfoxide, injected two days before tumor implantation) to pancreatic-cancer-bearing wildtype mice was compared against injections with solvent only (initially n = 10). The CCR4 antagonist AF399/420/18,025 was kindly provided by Prof. Dr. Jagadeesh Bayry (Paris, France) [37]. Third, the drug was tested in a therapeutic application scheme in the same model (initially n = 10). The first dose was administered two weeks after tumor inoculation, with additional doses (or vehicle controls) injected every four days until the experiments were terminated. After the second MRI, the animals were euthanized. Tumors were explanted, weighed, and prepared for histological analysis. For survival analysis, three experimental groups were generated as described previously (A: tumor-bearing CCR4-deficient mice, B: tumor-bearing WT mice with the prophylactic application of the CCR4 antagonist, C: tumor-bearing WT mice with the therapeutic application of the antagonist, control: tumor-bearing WT mice). Furthermore, mice of a different control group without implanted tumors were injected with the CCR4-antagonizing molecule to examine its potential toxic effects. The knockout group contained 22 mice, while the therapeutic and prophylactic groups each contained 20 mice. The control groups had 18–20 mice. In this examination, no further interventions were made. The death of the mice was controlled daily. To compare the composition of intratumoral immune cells in a CCR4-associated manner, tumors were implanted in wildtype mice and CCR4^−/−^ mice following the implantation procedure described previously. After five weeks, the animals were euthanized, and the tumors were processed for downstream analysis.

### 2.3. Magnetic Resonance Imaging (MRI)

The animals underwent MRI on days 21, 35, and/or 42 after tumor implantation to determine the tumor size and growth. The animals received isoflurane (1.0–1.5%) inhalation anesthesia, supervised by monitoring the animals’ respiratory rates. A sensor on each mouse’s thorax measured the respiratory rate, and the MR images were acquired in a breath-triggered manner. The mice were imaged by 7-Tesla small-animal MRI (ClinScan, 290 milli-tesla/m gradient strength) surrounded by a whole-body coil for better image quality and resolution (Bruker, Ettlingen, Germany). MRI imaging was performed in a T2-TSE (turbo spin-echo)-weighted sequence. The examination was conducted in the frontal and transverse planes. Volume determination was performed by evaluating MRI abdomen images using the MIPAV (Medical Imaging Processing, Analysis, and Visualization) algorithms (National Institutes of Health, Bethesda, MD, USA). Pancreatic tumors were marked as regions of interest. Computational algorithms determined the tumor volumes from the slice thickness of the images, the image resolution, and the regions of interest.

### 2.4. Immunohistochemistry

Paraffin-embedded tumors were sectioned onto microscopy slides. The sections were deparaffinized in full baths using xylene/methanol/ethanol. Primary anti-mouse F4/80 antibody (rat, monoclonal; Bio-Rad, Neuried, Germany) was diluted 1:50 in PBS containing 15% bovine serum albumin and incubated overnight at 4 °C on sections. The sections were washed, and peroxidase-conjugated secondary antibodies (anti-rat goat, polyclonal; Dianova, Eching, Germany) were diluted 1:200 in PBS containing 1% bovine serum albumin. The antibody-containing stain was applied on the sections for 1 h at room temperature. After washing, DAB (3,30-diaminobenzidine substrate kit SK4100; Vectorlabs, Newark, CA, USA) was added and incubated in a humid darkroom for 15 min. In addition, cells were stained with hematoxylin for approximately 1 min. Sections were capped with Kaiser’s glycerol gelatin. Then, 7–12 images of each section were acquired at 200× magnification (BZ9000; Keyence, Neu-Isenburg, Germany). The percentage of F4/80-positive cells (macrophages) was analyzed using IPAV (Centre for Information Technology; National Institutes of Health) or ImageJ (1.50i with Color Deconvolution extension; National Institutes of Health) software.

### 2.5. Flow Cytometry

Tumors were generated in wildtype and CCR4^−/−^ mice and explanted after 5 weeks. Five different subpopulations of intratumoral immune cells were quantified (F4/80^+^ macrophages, CD4^+^ T cells, CD8^+^ T cells, CD19^+^ B lymphocytes, and FOX-P3^+^ regulatory T cells/T_regs_). Before flow cytometry, the tumors were cut manually with scissors into pieces smaller than 1 mm and incubated (37 °C) with 2 mg/mL collagenase D and 4 mg/mL DNase I (both Roche, Mannheim, Germany) in HBSS buffer for 60 min in 15 mL falcon tubes, using a rotation robot. The tubes were vortexed every 15 min. The suspension was pressed through 100 µm cell sieves and 40 µm cell sieves for single-cell isolation using conventional 20 mL syringes. After washing the suspensions with HBSS buffer, unspecific binding was blocked using TruStain fcX (BioLegend, Amsterdam, the Netherlands) in FACS buffer. Cells were incubated with fluorescence-labeled monoclonal antibodies targeting CD4, CD19, CD8a, F4/80 (all BioLegend), and CD45 (Becton-Dickinson, Heidelberg, Germany). Afterward, the cells were permeabilized and fixed using FOXP3 staining buffer before intracellular staining of FOXP3 with fluorescence-labeled monoclonal antibody (BioLegend) was performed. Flow cytometry was performed using an LSR II device (Becton-Dickinson).

### 2.6. Migration Analysis

Murine macrophages were extracted from bone marrow from the hind legs of male CCR4^−/−^ and wildtype C57BL/6 mice. Cells were cultured and cultivated with 20 ng/mL M-CSF (Miltenyi Biotec, Teterow, Germany). On day 8, the cell supernatant was aspirated, and the cells were used for migration assays after checking cell viability via trypan blue assay. The cells showed a viability of at least 95%. Cell culture inserts with an 8 μm pore size for 12-well plates were used to perform migration assays. Here, 300 μL of Matrigel (1 mg/mL) was added to the inserts and incubated at 37 °C to induce polymerization. Unpolymerized Matrigel was removed. Then, 400 μL of penicillin- and streptomycin-supplemented cell culture medium containing 1 × 10^5^ murine macrophages from CCR4^−/−^ or wildtype C57BL/6 mice was added to the cell culture inserts (upper chamber). Cell culture supernatants from 6606PDA cells or fresh culture medium as controls were added to the lower compartment (culture dish wells) to serve as potential chemoattractants. After 12 h of incubation under cell culture conditions, migrated macrophages were isolated from the lower chamber using trypsinization and scratching before quantification (CyQUANT; Thermo Fisher Scientific, Dreieich, Germany).

### 2.7. Statistical Analysis

Statistical analysis was performed using Prism 9.4.1 (GraphPad Software; San Diego, CA, USA). Two independent groups were tested using the nonparametric Mann–Whitney test. Survival kinetics was examined by measuring the survival time from the day of tumor implantation and plotted using Kaplan–Meyer curves. To exclude animals that died prematurely due to postoperative complications rather than the implanted carcinoma, only mice surviving longer than two weeks after tumor implantation were included in analysis. Comparison of survival dynamics was performed using the Mantel–Cox test. The significance levels were set as follows: * = *p* < 0.05; ** = *p* < 0.01; *** = *p* < 0.001.

## 3. Results

### 3.1. CCR4 Inhibition and Depletion Were Associated with Lower Tumor Burden In Vivo

**Figure 1:**
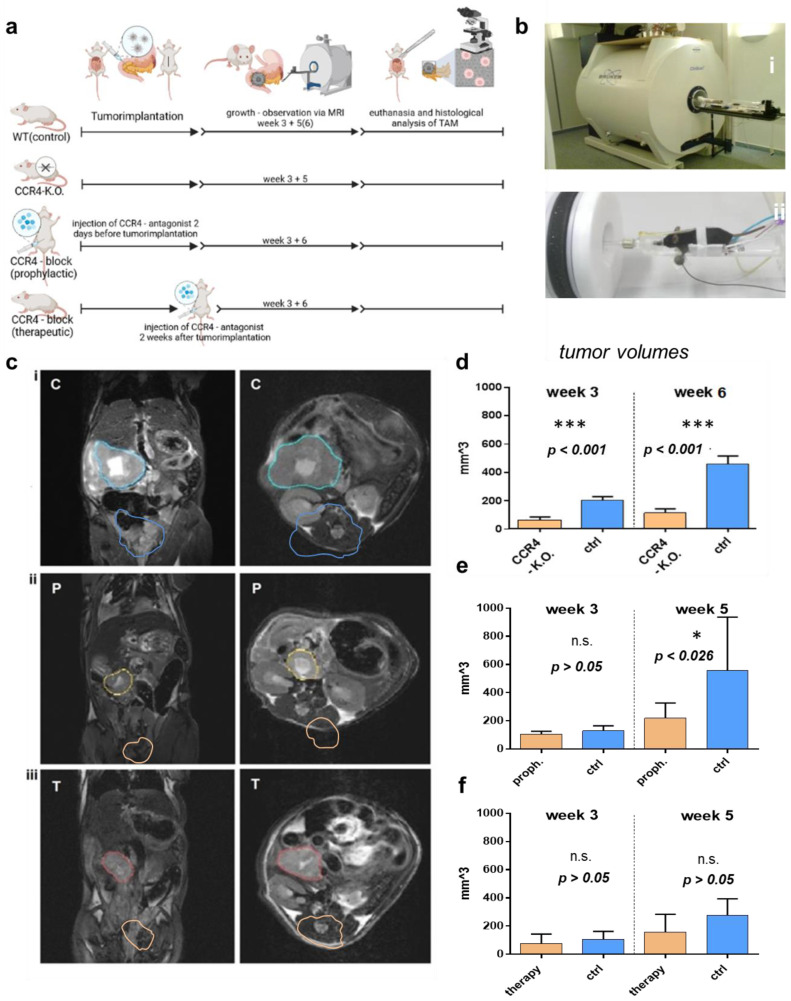
**Effects of CCR4 knockout/CCR4 antagonist on tumor growth.** (**a**) Settings of in vivo experiments: pancreatic carcinoma (PC) cells were implanted in the pancreas of wildtype mice as controls (C-group)* and CCR4^−/−^ mice (KO-group) the CCR4 antagonist (AF399/420/18025) was given to wildtype mice 2 days before PC implantation (P-group)*** or 2 weeks after tumor implantation (T-group); PC was implanted in wildtype mice as a control, DMSO was given instead of AF399/420/18025 in the T- and P-groups as a control, volumes were measured via MRI after 3 and 5 weeks in the P- and T-groups or after 3 and 6 weeks in the KO group. (**b**) Photograph of 7-tesla-PET magnetic resonance imager (**i**) and preparation of mice for imaging (**ii**). (**c**) MRI images for volume measurement in the sagittal (**left**) and transversal (**right**) dimensions; tumors are marked in blue (control) (**i**), yellow (P-group) with prophylactic CCR4-blockade (**ii**), and red (T-group) with therapeutic CCR4 blockade (**iii**). (**d**–**f**) Tumor volume in the CCR4^−/−^ group (KO) after 3 and 6 weeks (**d**), tumor volume in the CCR4 block (prophylactic) group (P) after 3 and 5 weeks (**e**), and tumor volume in the CCR4 block (therapeutic) group (T) after 3 and 5 weeks (**f**). The significance levels are indicated as follows: * = *p* < 0.05, *** = *p* < 0.001. n.s. = not significant.

In this study, three groups of C57BL/6 mice were used to investigate the effects of inhibiting CCR4 on tumor growth in pancreatic cancer in a syngeneic tumor model. The first group was a CCR4-knockout group that did not receive any pharmacological CCR4 inhibition before or after PDA6606 pancreatic cancer cells were implanted in the pancreas. The second group received prophylactic CCR4 inhibition before pancreatic cancer injection. The third group received CCR4 inhibition two weeks after tumor injection (Figure 1a). Wildtype mice with PC, which did not receive the CCR4 inhibitor, were used as controls. Magnetic resonance imaging (MRI) was used to monitor tumor growth at three to four weeks and five to six weeks after tumor initiation (Figure 1b). Tumor growth was assessed by quantitative volumetric measurements of MRI data (Figure 1c) for the three tumor models (Figure 1d–f). The exclusion criterion was premature death because of postoperative complications in the first 14 days after tumor implantation. Animals in which the tumor could not be delineated from the parenchyma on MRI because the size was too small were also excluded. In addition, a group of wildtype mice without PC was treated with the CCR4 antagonist in the same regime to rule out toxic effects. The results showed that genetic depletion or antagonization of CCR4 significantly decreased pancreatic cancer growth. After three weeks, the mean tumor volume in CCR4-knockout mice (73.2 ± 11.6 mm^3^ (n = 15) was significantly lower than in wildtype mice (204.2 ± 19.1 mm^3^ (n = 13)) (*p* < 0.001). After five weeks, the mean tumor size was also significantly smaller in CCR4^−/−^ mice (114.0 ± 19.2 mm^3^ (n = 11)) than in wildtype mice (442.1 ± 57.8 mm^3^ (n = 11)) (*p* < 0.001). In the second group (prophylactic pharmacological CCR4 inhibition before tumor implantation), antagonization of CCR4 significantly decelerated tumor growth (106.0 ± 20.5 mm^3^) compared to the vehicle control group (129.8 ± 35.1 mm^3^) (*p* = 0.317) after 3 weeks. Six weeks after tumor implantation, there was a significantly decreased tumor volume (219.7 ± 99.1 mm^3^) in mice receiving prophylactic CCR4 inhibition compared to vehicle controls (556.4 ± 351.8 mm^3^) (*p* = 0.026). In the third group (tumor-bearing mice receiving therapeutic pharmacological CCR4), CCR4 inhibition started 14 days after tumor implantation. The mean tumor volume at day 21 was significantly lower (77.4 ± 62.2 mm^3^) compared to vehicle controls (107.6 ± 51.7 mm^3^) (*p* = 0.130). On day 42, this difference was even higher (control: 274.1 ± 112.9 mm^3^; therapeutic antagonization: 157.5 ± 117.7 mm^3^; *p* = 0.092).

### 3.2. CCR4 Inhibition and Depletion Were Linked to Improved Tumor Survival In Vivo

**Figure 2:**
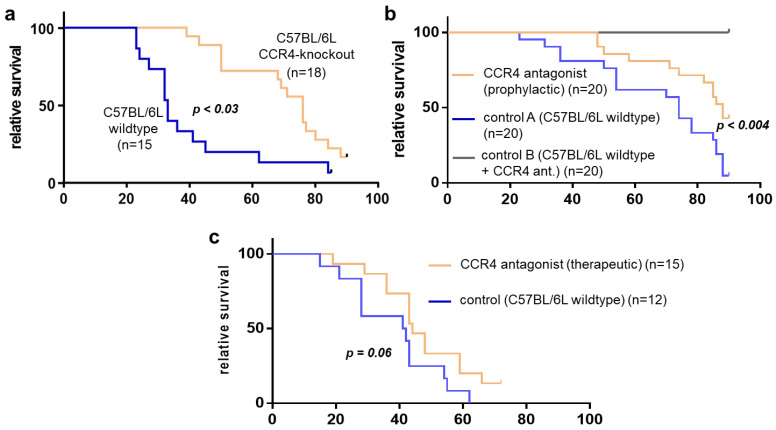
**Survival dynamics of CCR4-blocked mice with pancreatic cancer.** (**a**) Survival of CCR4-knockout mice with pancreatic cancer (PC)(orange) vs. controls (wildtype with PC). (**b**) CCR4-blocked mice with PC (prophylactic; orange) vs. controls (wildtype with PC; blue). (**c**) CCR4-blocked mice with PC (therapeutic; orange) vs. controls (wildtype mice with PC). The x-axis shows days.

Animal survival was analyzed for the three tumor models (Figure 2a–c). Pancreatic-cancer-bearing CCR4^−/−^ mice (tumor implantation n = 22; n = 5 died in <14 days; long-term survivors: n = 3; for statistical analysis: n = 14) benefited from significantly improved survival compared to their wildtype C57BL/6 counterparts (tumor implantation n = 18; n = 3 died in <14 days; long-term survivors: n = 1; for statistical analysis n = 14). The median survival (76 days) in CCR4^−/−^ mice (n = 14) was significantly enhanced (*p* = 0.004) compared to the control (33 days) group (n = 17), as also confirmed by the log-rank test. Similar findings were obtained in tumor-bearing mice that had received pharmacological CCR4 inhibition prior to tumor inoculation (tumor implantation n = 20, n = 0 died in <14 days; long-term survivors: n = 9) (median survival 88 days) compared to controls (tumor implantation n = 20; n = 0 died in <14 days; long-term survivors: n = 1) (median survival of 74 days) (*p* = 0.004) (Figure 2b). Injection of the pharmacological CCR4 inhibitor alone without tumor cell inoculation was well tolerated and was not fatal in any animal (n = 20) investigated over 90 days. The therapeutic application of the CCR4 inhibitor (tumor implantation: n = 20, n = 5 died in <14 days; long-term survivors: n = 2) gave a median survival of 44 days, which was not significantly improved (*p* = 0.066) compared to control animals (tumor implantation n = 20, n = 8 died in <14 days; long-term survivors n = 0) (median survival: 41 days) (Figure 2c). In summary, the experimental results showed that loss of the CCR4 chemokine receptor or antagonization of CCR4 in mice conferred a significant survival advantage in mice suffering from pancreatic cancer.

### 3.3. Loss of CCR4 Reduced Immune Cell Infiltration

**Figure 3:**
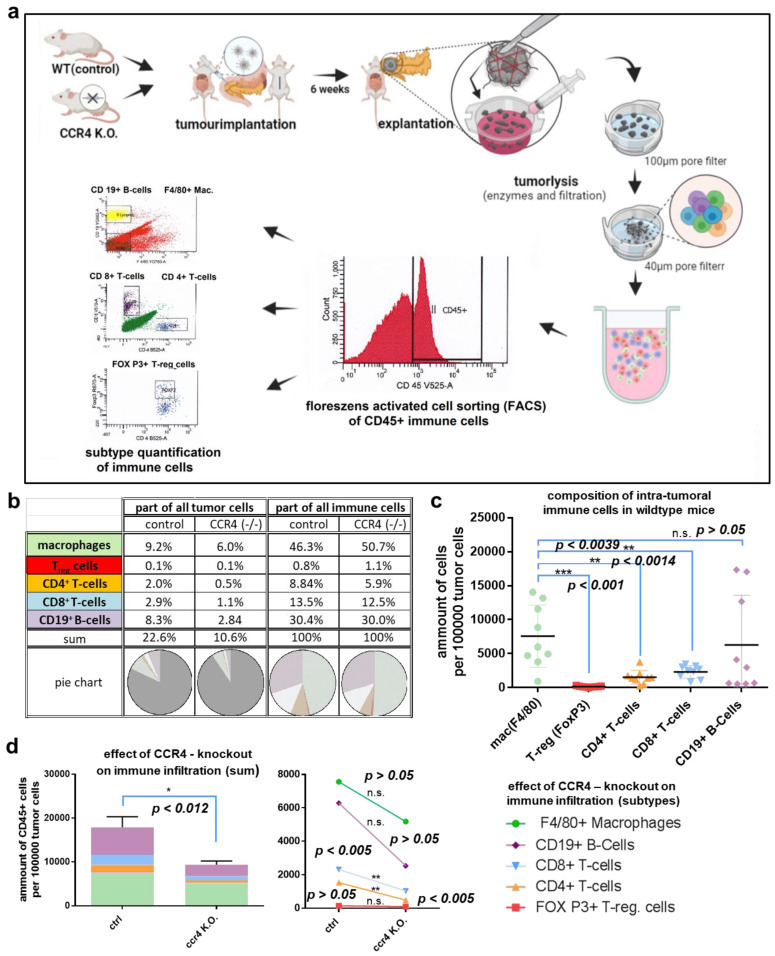
**Quantifying intratumoral immune cells via flow cytometry.** (**a**) Experimental settings: tumors were implanted in wildtype mice and CCR4^−/−^ mice as described, explantation was performed after 5 weeks, tumors were minced manually with scissors, collagenase, and DNase were used for tumor lysis, single-cell isolation was performed through 100 µm and 40 µm pore filters; intratumoral immune cells were detected via CD45 and subclassified to macrophages, B lymphocytes, CD4^+^ T cells, CD8^+^ T cells, and T_regs_ via antibody labeling and flow cytometry. (**b**) Composition of immune cells concerning all tumor cells (or immune cells only) in wildtype mice and CCR4^−/−^ mice (tabular). (**c**) Composition of intratumoral immune cells in wildtype mice. (**d**) CCR4 knockout effect on immune cell infiltration (**left**) and subtypes (**right**). The significance levels are indicated as follows: * = *p* < 0.05, ** = *p* < 0.01, *** = *p* < 0.001. n.s. = not significant.

To explore a potential immune-associated mechanism leading to improved survival in mice experiencing genetic CCR4 inhibition, pancreatic cancer tissue was lysed for single-cell isolation. Flow cytometry analysis revealed the quantitative composition of intratumoral immune cells of wildtype mice and CCR4^−/−^ mice (Figure 3a–c). The tumors of wildtype mice consisted of more than 22% immune cells on average. Macrophages comprised nearly 10% of the tumor cells (9.2% ± 1.8%). Thus, almost half of the immunological (CD45^+^) tumor infiltrate consisted of macrophages (46.7%). CD19^+^ B cells represented 8.3% ± 3.3% of tumor cells. All other examined immune subtypes were significantly less present in tumors of wildtype mice, such as CD4^+^ (1.9 % ± 0.5%) and CD8^+^ T cells (2.9% ± 0.4%). Regulatory T cells (T_regs_) represented the smallest proportion, at 0.8% ± 0.1.%. CCR4 knockout had a significant effect on tumor immune cell infiltration (CCR4^−/−^: 9341 ± 1777 CD45^+^ cells per 1 × 10^5^ tumor cells vs. WT: 17,860 ± 2462 CD45^+^ cells per 1 × 10^5^ tumor cells; n = 9, *p* = 0.0127). Thus, the number of intratumoral immune cells could be reduced by nearly 53% (Figure 3d). Considering the absolute decrease in the number of immune cells, B lymphocytes were most affected by the genetic loss of CCR4 (CCR4^−/−^: 2534 ± 809 CD19^+^ cells per 1 × 10^5^ tumor cells vs. WT: 6287 ± 2426 CD19^+^ cells per 1 × 10^5^ tumor cells; n = 9, *p* = 0.1616). Hence, on average, CCR4^−/−^ mice’s tumors contained 3753 ± 2557 less CD19^+^ B lymphocytes per 1 × 10^5^ tumor cells than the tumors of WT mice, corresponding to a relative reduction of 65%. Macrophages showed the second-largest CCR4-associated decline (CCR4^−/−^: 5180 ± 1524 F4/80^+^ cells per 1 × 10^5^ tumor cells vs. WT: 7563 ± 1524 F4/80^+^ cells per 1 × 10^5^ tumor cells; n = 9, *p* = 0.0127), with a mean absolute reduction of 2383 ± 2155 (35%). The number of tumor-infiltrating CD4^+^ T cells decreased significantly (CCR4^−/−^: 488 ± 72 CD4^+^ cells per 1 × 10^5^ tumor cells vs. WT: 1538 ± 355 CD4^+^ cells per 1 × 10^5^ tumor cells; n = 9, *p* = 0.0074) with a mean absolute reduction of 1050 ± 343 (72.5%). The number of CD8^+^ T cells decreased significantly as well (CCR4^−/−^: 1039 ± 263 CD8^+^ cells per 1 × 10^5^ tumor cells vs. WT: 2322 ± 307 CD8^+^ cells per 1 × 10^5^ tumor cells; n = 9, *p* = 0.0059), with a mean absolute reduction of 1283 ± 405 (59.7%). The absolute number of T_regs_ was hardly decreased (CCR4^−/−^: 100 ± 21 FOXP3^+^ cells per 1 × 10^5^ tumor cells vs. WT: 151 ± 29 FOXP3^+^ cells per 1 × 10^5^ tumor cells; n = 9, *p* = 0.175), with a mean absolute reduction of 51 ± 36, (42.1%).

### 3.4. CCR4 Inhibition and Depletion Affected Macrophage Migration and Infiltration

**Figure 4:**
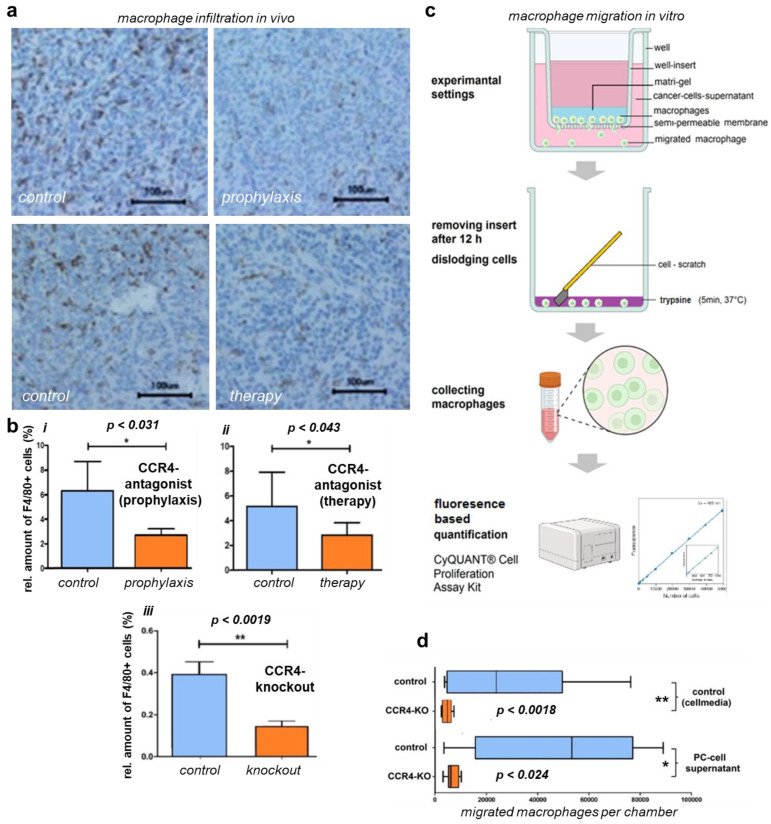
**CCR4 knockout/antagonist effects on macrophage migration and infiltration.** (**a**) Immunohistological tissue stains of in vivo tumors with mac receptor F4/80 show reduced infiltration of macrophages (mac) in the tumor after prophylactic CCR4 blockade and therapeutic treatment with CCR4 blockade compared to controls; CCR4^−/−^ tumors present similar results (histological stain not shown). (**b**) Quantification of F4/80-positive cells (alias macrophages), showing significantly reduced infiltration in CCR4-KO tumors. (**iii**); tumors of CCR4-antagonized mice also presented less macrophage infiltration in both the prophylactic and therapeutic groups (**i**,**ii**). (**c**) Workflow protocol of migration assay using Boyden chamber, murine intraperitoneal MCS-F-stimulated macrophages were placed in Matrigel and seeded in inserts, prior to the well bottoms being filled with PDA6606 supernatants as a migration stimulus; after 12 h of incubation, the insert was removed, and migrated cells were disengaged from the monolayer and collected for quantification via FACS. (**d**) In vitro migration of peritoneal macrophages of CCR4-KO-mice was reduced compared to wildtype macrophages. PC (peritoneal carcinomatosis)-cell-supernatant-stimulated macrophages presented increased migration compared to non-stimulated macrophages in both the CCR4 and wildtype groups. The significance levels are indicated as follows: * = *p* < 0.05, ** = *p* < 0.01.

Intratumoral macrophages contributed to the largest part of the intratumoral immune subpopulations. Since these cells are known for their pro-tumorous attributes, we investigated the intratumoral macrophages’ potential role in improved survival in mice experiencing pharmacological or genetic CCR4 inhibition (Figure 4a). For this purpose, pancreatic cancer tissue was harvested and sectioned. Immunohistochemical staining of the macrophage marker F4/80 suggested decreased macrophage numbers in the cancer tissue of CCR4^−/−^. Wildtype mice exposed to pharmacological CCR4 inhibition presented decreased macrophage numbers in tumor tissue compared to the tumors of control mice. Quantification of infiltrating macrophages from microscopy images revealed these differences to be significant in all three animal experiments performed in our study (Figure 4b). Specifically, histological sections of pancreatic carcinomas from CCR4^−/−^ mice showed 0.14 ± 0.02% of F4/80^+^ area compared to sections of tumors from C57BL/6 wildtype mice, which showed 0.39 ± 0.06% macrophage staining in sections from pancreatic cancer-bearing wildtype C57BL/6 mice (*p* = 0.002). In the CCR4 prophylaxis experiment, the percentage of F4/80^+^ area in the control group was 6.31 ± 1.07% on day 42 after tumor implantation, while in the prophylaxis group this percentage was 2.69 ± 0.26% (*p* = 0.031). In the animals receiving therapeutic CCR4 antagonist administration, an average of 2.85 ± 0.35% of the area was stained with F4/80^+^, compared to 5.14 ± 0.93% in the control animals (*p* = 0.043). To further understand CCR4-associated macrophage migration along chemotactic gradients, bone-marrow-derived macrophages were generated by in vitro stimulation with M-CSF. Subsequently, the cells were placed in dish inserts (upper chamber), and cell culture supernatants of 6606PDA cells were placed in the bottom chamber to serve as chemoattractants (Figure 4c). Macrophages from wildtype C57BL/6 control mice (n = 8) migrated to a significantly (*p* = 0.002) greater extent (49,230 ± 11,080) compared to macrophages from CCR4^−/−^ mice (n = 8; 6622 ± 839) (Figure 4d). Conventional culture medium was associated with an average migration of 29,030 ± 9669 from wildtype C57BL/6 macrophages, compared to 4635 ± 640 using CCR4^−/−^ macrophages (n = 8, *p* = 0.0246).

## 4. Discussion

This work aimed to investigate the role of the CCR4 chemokine receptor on pancreatic cancer progression in a murine orthotopic pancreatic cancer model. CCR4^−/−^ mice (genetic loss of CCR4 chemokine receptor) were compared with wildtype C57BL/6 mice (wildtype mice). The latter were compared against prophylactic or therapeutic pharmacologic CCR4 antagonization using the small molecule AF-399/420/18025. We identified significantly improved tumor volume and survival outcomes in animals with genetic or pharmacological CCR4 suppression, which correlated with decreased intratumoral macrophage numbers.

**Figure 5:**
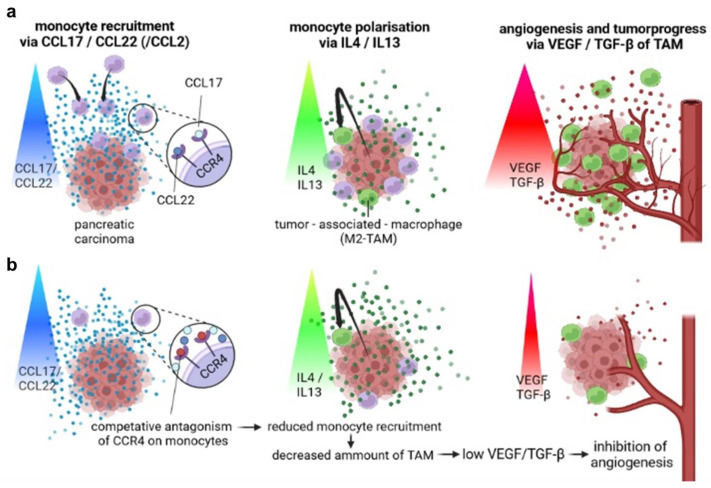
**Principle of the therapeutic concept of a CCR4 antagonist in pancreatic cancer.** (**a**) Monocyte recruitment via CCR4 ligands CCL17/CCL22 leads to TAM development and peritumoral angiogenesis. (**b**) Monocyte recruitment is inhibited by blocking CCR4 on monocytes. Consequently, monocyte infiltration and TAM development are decreased. Hence, angiogenesis and tumor progress are reduced.

Pancreatic carcinoma is known for its immunomodulatory capabilities (Figure 5). Macrophages and their precursors, monocytes, are attracted to the tumor and become polarized to tumor-associated macrophages (TAMs)—a pro-tumorous M2 phenotype. TAMs are a crucial part of the pancreatic TME and release soluble factors leading to immunosuppression, angiogenesis, metastasis, tumor growth, and poor prognosis [21,22,23,27,45,46]. Up to now, the CCL2 (monocyte chemoattractant protein 1) CCR2 axis has been seen as the main axis for macrophage recruitment and has already been targeted in clinical trials, with successful reduction in the numbers of circulating monocytes [24], although the long-term survival of patients with solid tumors has not been improved sufficiently yet [24,25]. To identify further potential and possibly more efficient strategies for TAM inhibition in this study, the role of the chemokine receptor CCR4 on TAM and its ligands CCL17 and CCL22 is of interest—especially in pancreatic cancer. It was recently shown that CCR4^+^ MDSCs (monocytic myeloid-derived suppressor cells) are linked to elevated EMT (epithelial–mesenchymal transition) in pancreatic cancer patients [47]. TAMs were reported to induce tumor progress via the CCR4-CCL17 axis in hepatocellular cancer, the CCR4-CCL22 axis in prostate cancer [33,34], and CCL22 in esophageal squamous-cell carcinoma [48].

To examine the association between CCR4 and TAMs in pancreatic cancer, we analyzed the immune cell abundance in lysed tumors of CCR4^−/−^ mice and wildtype mice via flow cytometry. We observed that macrophages represented the majority of the intratumoral immune cells, accentuating their relevance in the tumor microenvironment. A genetic loss of CCR4 reduced the infiltration of all examined immune cell subtypes, including macrophages. Immunohistochemical sections of pancreatic tumors revealed that intratumoral macrophages were significantly decreased in CCR4^−/−^ and CCR4-antagonized mice. This suggests CCR4 to be important for macrophage immigration in the TME, as underlined by our in vitro migration assay. The expression of CCR4 in macrophages has been known for a long time [2], and our data indicate its importance in tumor progression. Furthermore, high CCR4 expression in the TME correlates with poor prognosis in lung cancer patients [49]. Similarly, the results of our orthotropic pancreatic cancer model on CCR4^−/−^ mice showed that the loss of CCR4 reduces tumor growth and prolongs survival in pancreatic cancer in vivo.

Our experiments revealed a benefit of the CCR4 antagonist AF399/420/18025 injected prophylactically and trending therapeutically in pancreatic-cancer-bearing wildtype mice. The macrophage density in the implanted tumors was decreased upon CCR4 blockade. We suggest that reduced TAM infiltration, caused by the complete loss of CCR4 (CCR4^−/−^) or CCR4 blockade (antagonist), was responsible for the decreased tumor mass, according to the improved survival and reduced macrophages observed in our experiments, along with the previously reported pro-tumorous attributes of tumor-associated macrophages [21,22,23,27,45,46]. Direct modulation of TAM functionality via CCR4 blockade is also feasible. Berlato et al. showed that antagonizing CCR4 impacts the TAM phenotype in renal cancer [50]. TAMs of CCR4-blocked mice presented significantly higher levels of MHC class II and intracellular tumor necrosis factor, as well as lower levels of mannose-receptor and Arg1/*Nos2* expression ratios, indicating repolarization of M2 in favor of M1 in TAMs. Similar findings on the induction of M1 macrophages in the TME via CCR4 blockade were reported recently [51]. Therefore, suppressing M2 and triggering M1 macrophage phenotypes is a potential method to support immunological antitumor activity.

CD19^+^ B lymphocytes made up the second biggest share of all intratumoral cell types on average in our in vivo model. However, we observed a large variability in B-cell numbers in our experimental settings. In four explanted tumors, B cells were hardly represented. In contrast, three other tumors showed extraordinarily high B-cell numbers. We hypothesized that the reason for this was the accidental explantation of peritumoral lymph nodes, which were included in the flow cytometry analysis, so the significance of these results is limited. Nevertheless, the genetic loss of CCR4 led to a trend of reduced tumor-associated B-cell infiltration. We observed improved survival of CCR4^−/−^ mice with lower B-cell infiltration. This observation has no causal relationship, because B cells are associated with increased survival in pancreatic cancer [52,53]. Hence, we hypothesized a subordinate role for B lymphocytes in the survival of CCR4^−/−^ mice in our settings.

In addition to TAMs, regulatory T cells (T_regs_) are prominent pro-tumorous immune cells that deteriorate the prognosis in various malign entities [54,55]. T-regs’ impact on cancer progression has also been shown for pancreatic cancer [56]. Hence, T-regs have been qualified as a target for immunotherapy in cancer [57,58]. CCR4 in T cells has already been targeted in clinical trials with the anti-CCR4-antibody mogamulizumab in the therapy of cutaneous T-cell lymphoma. In this setting, the CCR4 blockade was shown to be a powerful method to inhibit the infiltration of T cells [56]. Chang et al. showed that the antitumor immunity of tumor-infiltrating T_regs_ can be forced via anti-CCR4 monoclonal antibodies in ovarian cancer in vivo [59]. Another Phase I trial by Doi et al. combining mogamulizumab with PD-1 inhibitors, showed a significant response to Mogamulizumab in 3 of 15 PC tumors [60]. Decreased rates of pro-tumorous T_regs_ and increased rates of anti-tumorous CD8^+^ cells in the tumors were promising. Physiologically, T_regs_ represent 4% of CD4^+^ cells, while they comprise 20–30% of the total CD4^+^ T cells in the tumor microenvironment [48], so they may possess a high potential as a therapeutic target. The genetic loss of CCR4 decreased T_reg_ infiltration by more than 40% in our experiments. This may have contributed to the improved survival of CCR4^−/−^ mice. However, in contrast to macrophages, T_regs_ were hardly represented in the tumors in our setting. This supports our hypothesis that the observed CCR4-linked anti-tumorous effects are mediated via TAMs. Although the degree of influence of immune cells of different entities generally cannot be equated with their quantitative presence, considering the huge numerical differences between macrophages and T cells, we believe that our hypothesis is quite valid. Furthermore, the CCR4 antagonist AF399/420/18,025, which was also used in the present study, did not induce a reduction in T-cell numbers in the tumor tissue of the murine pancreatic cancer model [61]. In a phase I clinical study on solid tumors, the anti-CCR4 antibody mogamulizumab showed no reduction in the numbers of immunosuppressing T_regs_ in pancreatic cancer [62]. Here, mogamulizumab did not show a significant therapeutic effect on solid tumors in this study.

Concerning therapeutic applications in patients, the potential anti-tumorous effects of pharmacological CCR4 blockade must be excluded for patients’ safety. All 20 control mice with no tumor but CCR4-block application survived until the end of the experiment, and abnormalities regarding behavior or vitality were not observed, so we can exclude direct and relevant toxic effects of CCR4 inhibition in our study.

Furthermore, we observed a dominant relative decrease in the numbers of CD4^+^ T cells and CD8^+^ T cells in CCR4^−/−^ compared to wildtype mice’s tumors. Considering the high expression of CCR4 in T cells, this result was not surprising [4]. Considering the anti-tumorous attributes of CD4^+^ and CD8^+^ T cells, this result needs to be critically evaluated [63,64].

It was recently shown that the CCL17-eluting scaffold prevents tumor progress in pancreatic cancer by attracting CCR4^+^CD4^+^ T cells [65]. Similarly, Rapp et al. showed that CCR4^+^ cytotoxic T cells decrease tumor growth because of CCL17/22 expressing PC properties and its microenvironment [66]. However, given the improved survival in our settings, as a hypothesis, we propose that the advantages of reducing pro-tumor macrophages and T_regs_ outweigh the possible disadvantages of reducing anti-tumorous T cells and B cells.

Considering that the cancer cells could have been affected directly by the pharmacological blockage of CCR4 in our experiments, which could be a valid explanation for the beneficial results. For instance, data from the “Protein Atlas” offer a lower survival in the later stages of PC, when tumor cells express high levels of CCR4 [67]. Furthermore, in malignant melanoma, CCR4 is expressed at higher levels in brain metastasis cells than in the primary cancer cells and is seen as a risk factor for metastasis. A previous study verified the association between CCR4 upregulation in human hepatocarcinoma and vascular invasion [47]. Targeting of CCR4 decreased cancer cell migration and metastasis in head and neck cancer [68]. However, in our CCR4^−/−^ model, PDA6606 tumor cells were not affected directly by the genetic knockout of CCR4 because they were implanted, but a benefit of CCR4 loss on survival in non-tumoral cells was shown. This suggests that the effects are not due to a direct action on the tumor cell but are indirectly mediated, which suggests an immunological influence.

As ancillary findings, it was striking that the control mice presented a variety of survival in the three in vivo experiments. We suspect that this was due to the use of implanted PDA cells of different passages between the experiments, which may have altered the proliferation rates or prompted a biological bottleneck effect caused by cell splitting, considering that the other experimental conditions were constant. Nevertheless, the PDA properties were the same within the individual experiments between the control and CCR4^−/−^ groups or CCR4-antagonistic groups. The methods for detecting macrophages (flow cytometry and immunohistochemistry) revealed the same trends with regard to the effects of a loss of CCR4, but they showed different proportions of macrophages in the tumor. This could be explained by the inclusion of peritumoral tissue containing macrophages in flow cytometry and the higher sensitivity of flow cytometry in macrophage detection compared to immunohistochemistry [69].

In addition to chemokine-associated immune recruitment as a pro-tumorous mechanism, pancreatic ductal adenocarcinoma (PDAC) is known for its ability to evade the immune system using different strategies, allowing the tumor cells to proliferate and spread. Recent studies have shed light on the immune evasion mechanisms employed by PDAC cells, including intrinsic and extrinsic factors that suppress the immune response. Some of the intrinsic factors include loss of major histocompatibility complex (MHC) expression, upregulation of immune-checkpoint proteins (e.g., PD-L1), and activation of oncogenic signaling pathways (e.g., KRAS), among others [69,70,71]. Extrinsic factors include the recruitment of immunosuppressive cells, such as myeloid-derived suppressor cells (MDSCs) and regulatory T cells (Tregs), along with the secretion of immunosuppressive factors, such as transforming growth factor-beta (TGF-β), interleukin-10 (IL-10), and VEGF [69,72,73,74].

Other CCR ligands are also involved in this cancer. It is known that CCR2, which is highly expressed in monocytes, is a crucial trigger for monocyte recruitment via the CCR2 ligand CCL2 [75]. In a previous study, we showed that pancreatic cancer cells (murine cell line PDA6606) release high amounts of CCL2 [27]. Furthermore, it has been reported that monocyte mobilization via the CCL2/CCR2 axis decreases survival in pancreatic cancer [76]. Consequently, the CCL2–CCR2 axis is also discussed as a potential target for immunotherapy in pancreatic cancer. CCL4, also known as macrophage inflammatory protein (MIP)-1β, was reviewed with respect to tumor progression. CCL4 (the ligand of chemokine CCR5) plays a critical role in the enrolment of regulatory T cells and pro-tumorigenic macrophages. Its influence on other tumor microenvironment cells—such as fibroblasts and endothelial cells—is also described as pro-tumorous [77]. Another CCR5 ligand is CCL5. CCR5 is expressed in pancreatic cancer cells. The interaction between CCR5 and CCL5 results in increased migration and invasiveness of pancreatic cancer cells. This axis should be explored in terms of its potential to inhibit metastasis in pancreatic cancer [78].

In addition to tumor cells, immune cells can have an impact on macrophage polarization and the development of TAMs. The interaction between immune cells is crucial in shaping the tumor microenvironment. Tumor-infiltrating T_H_1 cells secrete IFN-γ and TNF-α, which activate macrophages toward the pro-inflammatory M1 phenotype [79]. Conversely, T_H_2 cells secrete IL-4 and IL-13, which induce the development of pro–tumorous M2 macrophages [80]. Another interleukin for M2 polarization is IL-10, secreted by T_regs_, which is known to have a pro-tumorous character [81]. Dendritic cells (DCs) can recruit macrophages to the tumor via CCL2 and CCL5. The interaction between dendritic cells and macrophages is complex. DCs secrete M1-inducing IL-12 and TNF-α, along with M2-inducing IL-10 and TGF-β, so DCs have heterogeneous effects on macrophage polarization.

Recent advances in immunotherapy have led to the development of immune-checkpoint inhibitors (ICIs), which have shown promising results in the treatment of various cancers, including PDAC [82] ICIs work by blocking the interaction between immune-checkpoint proteins (e.g., PD-L1) and their receptors on T cells, thereby releasing the immune system’s brakes and allowing it to attack cancer cells. Although ICIs have shown some efficacy in clinical trials, the response rates in PDAC patients have been relatively low, and many patients have developed resistance to these therapies [83].

To overcome these limitations, researchers are exploring new strategies that combine ICIs with other treatments, such as chemotherapy, radiation therapy, and targeted therapy. For example, preclinical studies have shown that combining ICIs with gemcitabine—a standard chemotherapy drug used for PDAC—can enhance the antitumor immune response and improve survival in mouse models. Other preclinical studies have shown that targeting the tumor microenvironment, such as by depleting MDSCs or blocking TGF-β signaling, can also enhance the efficacy of ICIs [84,85,86].

In addition to ICIs, other immunotherapeutic approaches are also being investigated for treating PDAC, such as cancer vaccines, adoptive T-cell therapy, and oncolytic viruses. These approaches aim to stimulate the immune system to recognize and attack cancer cells, either by targeting specific tumor antigens or by introducing immune-stimulating factors directly into the tumor microenvironment [87,88,89,90]. Targeting immune evasion mechanisms in PDAC is a promising approach for developing more effective therapies for this aggressive cancer. By combining immunotherapy with other treatments and targeting both the genetic and microenvironmental factors simultaneously, we may overcome the limitations of current PDAC treatments and improve patient outcomes.

Our study had two limitations: First, murine cancer models reflect human pathophysiology only to a limited extent, but human tissues and clinical investigations were beyond the scope of this study. Second, we only used one cell line, and the results might diverge when using other syngeneic, orthotopic pancreatic cancer models.

## 5. Conclusions

This study identified the relevance of CCR4 on TAMs in pancreatic cancer and its therapeutic potential to target TAMs. Genetic loss of CCR4 reduced TAMs and T_regs_ as well as T cell numbers. These contrasting effects of CCR4 blockade could explain the unsatisfying results of clinical trials with CCR4 antagonists. Based on our results, we suggest that antagonizing CCR4 could be beneficial as an adjuvant strategy in pancreatic cancer therapy to mitigate the immunomodulatory potential of the remaining cancer cells after curative surgery and prolong the relapse-free period. Synergistic effects with CCR2 inhibitors should be investigated in future in vivo studies.

## Data Availability

The underlying data of this research can be received from the corresponding authors upon reasonable request.
